# Does beta-alanine supplementation enhance adaptations to resistance training? A randomized, placebo-controlled, double-blind study

**DOI:** 10.5114/biolsport.2023.112967

**Published:** 2022-03-16

**Authors:** Julio Benvenutti Bueno de Camargo, Felipe A. Brigatto, Rafael S. Zaroni, Moises D. Germano, Darlan Souza, Reury F. Bacurau, Paulo H. Marchetti, Tiago V. Braz, Marcelo S. Aoki, Charles R. Lopes

**Affiliations:** 1 Methodist University of Piracicaba, Human Performance Research Laboratory, Piracicaba, São Paulo, Brazil; 2 School of Arts, Sciences and Humanities, University of São Paulo, São Paulo, Brazil; 3 California State University Northridge, CA, USA

**Keywords:** Supplement, Carnosine, Morphology, Strength training

## Abstract

The aim of the present study was to assess the effects of beta-alanine supplementation on muscle strength and thickness. Nineteen resistance-trained men (age: 27.3 ± 5.5 years; height: 178 ± 10 cm; body mass: 83.4 ± 9.7 kg; training experience: 5.9 ± 3.9 years) were allocated to one of the following groups: Beta-alanine (BA) (6.4 g/day of beta-alanine) or Placebo (PLA) (6.4 g/day of maltodextrin). Subjects completed 4 resistance training sessions per week for 8 weeks. The following assessments were performed before and after intervention periods: 1 repetition maximum (1RM) and 60%1RM tests in the bench press (60%1RM_BENCH_) and back squat (60%1RM_SQUAT_) exercises; muscle thickness assessment of biceps brachialis (MT_BB_), triceps brachialis (MT_TB_)_,_ and vastus lateralis (MT_VL_) by ultrasonography. No significant difference between groups was observed for the absolute increase (pre-post intervention) in the 1RM_BENCH_ (mean difference = 0.8 kg; p = 0.679), 1RM_SQUAT_ (mean difference = 0.1 kg; p = 0.992), MT_BB_ (mean difference = 0.7 mm; p = 0.637), MT_TB_ (mean difference = 1.4 mm; p = 0.282), MT_VL_ (mean difference = 1.6 mm; p = 0.311), 60%1RM_BENCH_ (mean difference = 0.5 repetitions; p = 0.670) and 60%1RM_SQUAT_ (mean difference = 0.7 repetitions; p = 0.690). In conclusion, the 8-week training period induced significant strength and morphological responses. However, the addition of beta-alanine supplementation did not enhance these adaptive outcomes.

## INTRODUCTION

Anaerobic glycolysis is the predominant energy source during high-intensity exercise, which induces increased intracellular accumulation of hydrogens ions (H ^+^) [1]. T[his phenomenon, in turn, may result in decreased intramuscular pH, leading to muscle fatigue and, consequently, impaired neuromuscular performance [2]. Therefore, improving the cell’s ability to buffer H ^+^ may offer an advantage in the management of fatigue and a subsequently improved capacity to perform high-intensity activities. Consequently, sports practitioners and researchers often test different ergogenic aids (e.g., sodium bicarbonate and beta-alanine [BA]) that could enhance the performance and adaptive responses during high-intensity exercises by providing an augmented buffering potential.

Carnosine is an intracellular dipeptide located in the cytoplasm, especially in skeletal muscle cells, where it is synthesized from the amino acids L-histidine and BA [3, 4]. Higher muscle carnosine content has been shown to attenuate fatigue during exhaustive dynamic exercise [5] and induce higher calcium sensitivity of the contractile apparatus [6]. However, carnosine synthesis is limited by BA availability, since skeletal muscle cells have a high L-histidine content [4]. The chronic supplementation of BA between 4 and 24 weeks appears to be safe [7] and can increase skeletal muscle carnosine content by up to 200% [8], with strong evidence supporting the ergogenic role of supplementation in exercises ranging from 30 s to 10 min in duration [9].

Due to the anaerobic nature of resistance training (RT), one can suggest that BA supplementation may present a potential ergogenic aid for practitioners. Most studies that adopted a supplementation protocol alongside RT programmes assessed maximal strength, power, and strength endurance outcomes [10, 11]. However, to our knowledge, no previous study has investigated the effects of BA on morphological adaptations of specific muscles, with only indirect measures of total lean mass (e.g. whole-body dual-energy X-ray absorptiometry) being adopted to the present date [10, 12, 13]. Therefore, the present study aimed to assess the chronic effects of BA supplementation on strength and hypertrophy outcomes in RT men. Our initial hypothesis was that the supplementation protocol adopted would maximize both strength and morphological adaptations induced by the RT programme.

## MATERIALS AND METHODS

### Participants

**FIG. 1:**
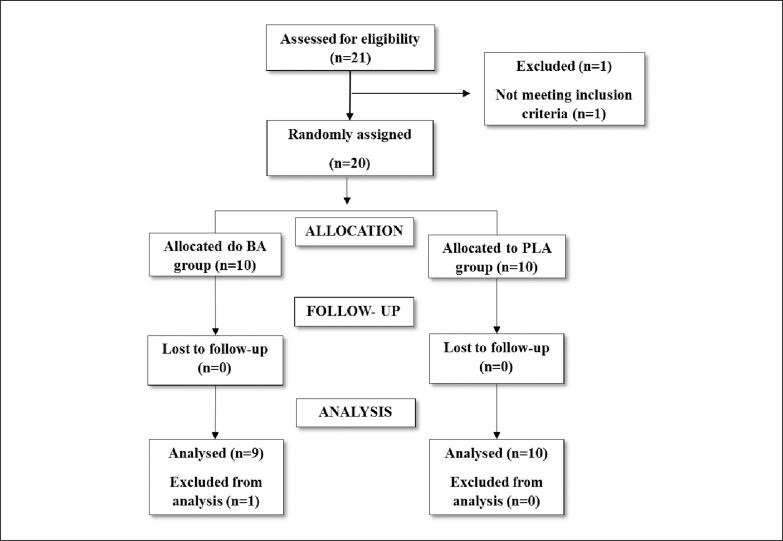
Subject recruitment and flow through the protocol. BA (beta-alanine); PLA (placebo).

Twenty healthy males (age: 27.4 ± 5.3 years; height: 178 ± 10 cm; total body mass: 83.4 ± 9.7kg; RT experience: 5.9 ± 3.9 years) who were recruited from a resistance-trained population participated in the study. A minimum final sample size of 20 participants was required according to a priori power analysis where the vastus lateralis muscle thickness was assessed as the outcome measure with a target effect size difference of 0.75, an alpha level of 0.05 and a power (1−*β*) of 0.80 [14]. Volunteers performed RT (minimum frequency of once a week) and all exercises adopted in the training intervention and the strength tests for at least 1 year before entering the study. In addition, participants stated that they were not consuming any dietary supplements that could enhance performance for a minimum of 6 months before the start of the study and presented relative one repetition-maximum (1RM) values of 1.25x and 1x total body mass in parallel back squat and bench press exercises, respectively [15]. Although 20 volunteers initiated the experiment, 1 from the BA group was excluded from the analysis due to a training frequency lower than 85%. Therefore, BA and PLA groups had 9 and 10 subjects, respectively (Figure 1). This study was approved by a university research ethics committee (protocol 2.094.534) and was conducted in accordance with the Declaration of Helsinki. All subjects read and signed an informed consent document.

### Experimental protocols

The study followed a randomized, placebo-controlled, double-blind design [16]. Participants were matched for strength, based on their relative one maximum repetition (1RM) bench press and parallel back squat scores, and subsequently randomly allocated to receive either beta-alanine (BA) or placebo (PLA) supplementation. The experimental period lasted 10 weeks: 1^st^ week – familiarization period and pre-intervention assessments (baseline); 2^rd^-9^th^ weeks – training and supplementation intervention period; 10^th^ week – post-intervention assessments. Assessments of maximal dynamic muscle strength (1RM test for bench press and parallel back squat exercises), muscular endurance (maximum repetitions at 60% of 1RM test for bench press and parallel back squat exercises), and muscle thickness (ultrasound) were performed at both pre- and post-intervention time points.

### Supplement schedule

Participants received either β-alanine (99.9% pure β-alanine, non-sustained release formula; CarnoSyn, NAI, San Marcos, California, USA) or a placebo (maltodextrin, Neonutri, Pocos de Caldas, Minas Gerais, Brazil) 4 times per day (4 doses of 1.6 g each [total of 6.4 g/day]), separated by 3–4 hours during the entire training intervention period (8 weeks) on both training and non-training days. This dosage was adopted based on previous studies that reported increases in muscle carnosine content following BA supplementation [10]. The capsules for both groups were similar in appearance, weight (400 mg), and taste. Enough supplement for 8 weeks was provided in an unlabelled and sealed pot separated by a researcher not involved in data collection. Adherence was determined by counting the amount of supplemental capsules remaining at the post-supplementation period. A minimum of 85% compliance was required [11]. Any side-effects of supplementation (e.g., paraesthesia) were individually monitored during the study. If a participant forgot to take a dose, an additional dose in another period or on a different day was to be ingested to complete the total dose of 358.4 g by the end of the study.

### Resistance training procedures

Before the training intervention period, all participants underwent 12-repetition maximum (12RM) testing (according to guidelines established by the National Strength and Conditioning Association [NSCA] [17]) to determine individual initial training loads for each exercise.

The RT protocol adopted was the same for both groups. During each intervention week, participants performed 4 weekly split routine sessions (A – Mondays and Thursdays; B – Tuesdays and Fridays); (pectoralis major, deltoids and triceps brachii in routine A; quadriceps, latissimus dorsi, and biceps brachii in routine B). Three sets were performed per exercise with a corresponding load of 12 RM, with 60 seconds of rest between sets and 120 seconds between exercises [18]. Participants were instructed to perform each set to the point of momentary concentric muscular failure. If more than 12 repetitions could be performed in the last set of a given exercise, increments in the external load ranging from 5% to 10% were implemented in the next training session. Training routines were supervised by the research team to monitor the proper technique and ensure the safety of the subjects.

### Food intake estimation

To avoid potential confounding of outcomes induced by diet, subjects were instructed to maintain their usual nutritional regimen and to avoid taking any supplements during the intervention period. A 24-hour food recall on 2 non-consecutive weekdays and 1 day at the weekend was adopted to assess dietary nutrient intake [19], which was analysed through NutWin software (UNIFESP, São Paulo, Brazil).

### Muscle thickness (MT)

Measurements of MT were obtained through ultrasound imaging. All testing was performed by a trained technician through the use of an A-mode ultrasound imaging unit (Bodymetrix Pro System; Intelametrix Inc., Livermore, CA, USA). A 2.5-MHz linear array ultrasound probe was placed perpendicular to the tissue interface without depressing the skin. High-quality images were saved to the hard drive and MT dimensions were obtained by measuring the distance from the subcutaneous adipose tissue–muscle interface to the muscle–bone interface [20]. Measurements of 3 sites were taken on the right side of the body: biceps brachii (MT_BB_), triceps brachii (MT_TB_), and vastus lateralis (MT_VL_). The upper arm measurements were made while subjects were standing, and the thigh muscle measurements were taken through a lying supine position.

For MT_BB_ and MT_TB_, measurements were obtained 60% distal between the lateral epicondyle of the humerus and the acromion process of the scapula. For MT_VL_, measurements were taken at 50% of the distance between the lateral condyle of the femur and the greater trochanter. A henna ink mark (reinforced every week) was used to maintain consistency between pre and post-intervention testing. To ensure that MT values were not overestimated from muscle swelling, images were obtained at least 48 h after training sessions, for both pre- and post-intervention assessments [21].

Additionally, for each site, at least 3 images were obtained to ensure the accuracy of measurements. According to the methodology described by Brigatto et al. [15], if measurements were within 1 mm of one another the figures were averaged to obtain a final value. If measurements were more than 1 mm from one another, a fourth image was obtained, and the closest 3 measurements were then averaged. The test-retest intraclass correlation coefficient (ICC) values from our laboratory for MT_TB_, MT_BB,_ and MT_VL_ were 0.998, 0.996, and 0.999, respectively. The coefficient of variation (CV) for these measures were 0.6, 0.4, and 0.6%, respectively. The standard error of the measurement (SEM) for these measures was 0.42, 0.29, and 0.41 mm, respectively.

### Muscle strength

Upper- and lower-body maximum strength was assessed by 1RM testing in the bench press (1RM_BENCH_) and parallel back squat (1RM-_SQUAT_) exercises. Subjects reported to the laboratory having refrained from any exercise at least 48 hours before baseline and post-intervention assessments. 1RM testing was consistent with recognized guidelines, as established by the NSCA [17]. The ICC, CV and SEM from our laboratory for 1RM_SQUAT_ were 0.989, 0.8% and 2.05 kg, respectively. The ICC, CV and SEM for 1RM_BENCH_ were 0.990, 0.7% and 1.95 kg, respectively.

### Muscle endurance

Ten minutes after 1RM tests, participants performed as many repetitions as possible until muscle failure at 60% of 1RM load [22] on both the bench press (60%1RM_BENCH_) and parallel back squat (60%1RM_SQUAT_). A standardized cadence of 40 bpm was adopted for each assessment (Metronome Beats, Stonekick). The ICC, CV and SEM for 60%1RM_SQUAT_ from our laboratory were 0.910, 3.3% and 1.13 repetitions, respectively. The test-retest ICC, CV and SEM for 60%1RM_BENCH_ were 0.943, 2.3% and 0.83 repetitions, respectively

### Total load lifted (TLL)

TLL (sets × repetitions × external load [23]) was calculated from training logs filled out by the researchers for every RT session. The weekly TLL was calculated as the sum of all loads lifted during the RT sessions that week. Additionally, the ∆TLL described the difference in the TLL between weeks 8 and 1 (e.g., TLL in week 8 minus TLL in week 1). The data were expressed in kilogram-force units (kgf).

### Statistical analysis

The normality and homogeneity of the variances were verified using the Shapiro-Wilk and Levene tests, respectively. The mean, standard deviation (SD) and 95% confidence intervals (CI) were used after the data normality was checked. To compare mean values of the descriptive variables, food intake variables, total capsules consumed, total RT sessions performed, ΔTLL week 8 minus week 1 between groups (BA vs PLA), and the absolute difference (pre-to-post changes) between groups in MT, 1RM and 60% 1RM, an unpaired t-test was used. A 2x2 repeated measures analysis of variance (ANOVA) (interaction groups [BA and PLA] × time [pre- vs post-intervention]) was used to compare the dependent variables (1RM_BENCH_, 1RM_SQUAT_, 60%1RM_BENCH_, 60%1RM_SQUAT_, MT_TB_, MT_BB_, and MT_VL_). Post hoc comparisons were performed with the Bonferroni test. Mauchly’s test of sphericity was applied and the Greenhouse-Geisser epsilon correction was used when the sphericity criteria were not met. Effect sizes were evaluated using partial eta squared (η^2^*p*), with < 0.06, 0.06–0.14 and, > 0.14 indicating a small, medium, and large effect, respectively [24]. The adopted significance was 5%. Effect sizes were also estimated for pairwise comparisons using Cohen’s d. The *d* results were qualitatively interpreted using the following thresholds: < 0.2, trivial; 0.2–0.6, small; 0.6–1.2, moderate; 1.2–2.0, large; 2.0–4.0, very large and; > 4.0, nearly perfect. Data analysis was performed using a modified statistical Excel spreadsheet [24] and SPSS-22.0 software (IBM Corp., Armonk, NY, USA). The figures were formatted in GraphPad Prism version 6.0 software (La Jolla, CA, USA).

## RESULTS

No significant difference was observed between groups for any baseline measurement (Table 1). No significant difference was noted between groups for total capsules consumed (873.3 ± 44.9 [97.4 ± 5.0% of compliance] vs 850.4 ± 44.9 [94.5 ± 6.2% of compliance], BA vs PLA, respectively) and total training sessions performed (30.6 ± 1.1 vs 30.6 ± 1.7, BA vs PLA, respectively) during the intervention period. Three out of 9 participants correctly guessed that they were taking BA, while 3 of 10 participants correctly guessed that they were ingesting a placebo.

**TABLE 1:** Baseline descriptive statistics for the BA and PLA groups (mean ± SD).

Variables	PLA (n = 10)	BA (n = 9)	*P value*
Age (years)	28.5 ± 5.5	26.1 ± 5.5	0.311
Body Mass (kg)	86.9 ± 11.1	81.2 ± 6.0	0.082
Height (cm)	177 ± 10	178 ± 10	0.472
RT experience (years)	6.1 ± 4.7	5.9 ± 4.7	0.392
RT frequency (sessions · wk^-1^)	5.0 ± 0.1	4.6 ± 0.5	0.583
Total number of sets (sets · wk^-1^)	91.8 ± 5.4	91.6 ± 4.3	0.915
Total energy intake (kcal)	2401 ± 339	2322 ± 300	0.762
Protein intake (g.kg^-1^)	1.6 ± 0.3	1.7 ± 0.3	0.823
Carbohydrate intake (g.kg^-1^)	4.1 ± 0.7	4.3 ± 0.8	0.934
Fat intake (g.kg^-1^)	0.5 ± 0.1	0.5 ± 0.1	0.928

PLA = placebo group; BA = beta-alanine group; RT = resistance training; sessions · wk^-1^ = sessions per week; sets · wk^-1^ = sets per week; kcal = kilocalories; g/kg^-1^ = grams per kilogram of body mass.

### Muscle thickness

A significant main effect of time (*F*_1,17_ = 11.807, *p* = 0.003, η^2^*p* = 0.410), but not group × time interaction (*F*_1,17_ = 0.216, *p* = 0.648, η^2^*p* = 0.013), was observed for MT_BB_. No significant between-group difference was noted for the absolute increase in MT_BB_ (pre- to post-intervention) (mean difference [95%CI] = 0.7 [-2.3 to 3.7 mm]; *p* = 0.637). The between-group effect size was small (*d* = 0.22) (Table 2).

**TABLE 2:** Pre and post 8 weeks muscle morphology measures for the BA and PLA groups (mean ± SD).

Variables	Pre	Post 8 weeks	MD [95%CI]	time *P* value	time*group *P* value
MT_BB_ (mm)					
PLA	42.2 ± 8.6	45.0 ± 6.8*	2.8 [0.7 to 4.9]	0.011	0.648
BA	44.7 ± 4.5	46.9 ± 5.4	2.2 [-0.6 to 4.3]	0.056	

MT_TB_ (mm)					
PLA	39.1 ± 5.1	42.1 ± 4.1*	3.0 [1.1 to 4.9]	0.003	0.279
BA	39.7 ± 5.4	41.3 ± 5.1	1.6 [-0.3 to 3.5]	0.105	

MT_VL_ (mm)					
PLA	46.5 ± 9.9	50.8 ± 8.7*	4.3 [2.0 to 6.5]	0.001	0.315
BA	45.5 ± 6.3	48.2 ± 6.0*	2.7 [0.2 to 5.0]	0.030	

BA = beta-alanine group; PLA = placebo group; MT_BB_ = muscle thickness of the biceps brachii muscle; MT_TB_ = muscle thickness of the triceps brachii muscle; MT_VL_ = muscle thickness of the vastus lateralis muscle; MD = Mean Difference; CI = Confidence Interval.

* p < 0.05 vs pre-intervention.

For MT_TB_, only a significant main effect of time (*F*_1,17_ = 13.011, *p* = 0.002, η^2^*p* = 0.434), but not group × time interaction (*F*_1,17_ = 1.253, *p* = 0.279, η^2^*p* = 0.069), was noted. No significant difference between groups was observed for the absolute increase (pre- to post-intervention) (mean difference [95%CI] = 1.4 [-1.2 to 4.1 mm]; *p* = 0.282). A small effect size (*d* = 0.53) between groups was revealed (Table 2).

A significant main effect of time (*F*_1,17_ = 19.899, *p <* 0.001, η^2^*p* = 0.539), but not group × time interaction (*F*_1,17_ = 1.073, *p* = 0.315, η^2^*p* = 0.059), was found for MT_VL_, while no significant difference between groups was found for the absolute increase (mean difference [95%CI] = 1.6 [-1.6 to 4.9 mm]; *p* = 0.311). The between-group effect size was small (*d* = 0.49) (Table 2).

### Maximal strength

For 1RM_BENCH_, only a significant main effect of time (*F*_1,17_ = 40.062, *p* < 0.001, η^2^*p* = 0.702), but not group × time interaction (*F*_1,17_ = 0.177, *p* = 0.679, η^2^*p* = 0.010), was revealed. For the absolute increase in 1RM_BENCH_, no significant between-group difference was found (mean difference [95%CI] = 0.8 [-3.3 to 4.9 kg]; *p* = 0.679). A small effect size (*d* = 0.20) was observed between groups (Table 3).

**TABLE 3:** Pre- vs. post-intervention muscle strength and muscle endurance measures for the BA and PLA groups (mean ± SD).

Variables	Pre	Post 8 weeks	MD [95%CI]	time*group *P-value*
1RM_BENCH_ (kg)				
PLA	109.8 ± 12.7	116.4 ± 10.6 ^Ɨ^	6.6 [3.7 to 9.4]	0.679
BA	106.7 ± 11.8	112.4 ± 11.7 *	5.7 [2.7 to 8.7]	

1RM_SQUAT_ (kg)				
PLA	108.6 ± 11.8	127.2 ± 20.7*	18.6 [8.0 to 29.1]	0.993
BA	106.4 ± 15.2	125.1 ± 20.7*	18.7 [7.5 to 29.8]	

60%1RM_BENCH_ (rep)				
PLA	15.1 ± 1.7	17.2 ± 1.5*	2.1 [0.6 to 3.5]	0.671
BA	16.8 ± 2.5	18.4 ± 2.5*	1.6 [0.1 to 3.2]	

60%1RM_SQUAT_ (rep)				
PLA	19.1 ± 7.8	22.7 ± 6.0*	3.6 [1.0 to 6.1]	0.690
BA	19.1 ± 5.2	22.0 ± 4.4*	2.9 [0.2 to 5.5]	

PLA = placebo group; BA = beta-alanine group; 1RM_BENCH_ = one repetition maximum test in the bench press exercise; 1RM_SQUAT_ = one repetition maximum test in the back squat exercise; 60%1RM_BENCH_ = 60% of 1RM test in the bench press exercise; 60%1RM_SQUAT_ = 60% of 1RM test in the back squat exercise; MD = Mean Difference; CI = Confidence Interval.

* p < 0.05 vs pre-intervention;

Ɨ p < 0.001 vs pre-intervention.

A significant main effect of time (*F*_1,17_ = 26.225, *p* < 0.001, η^2^*p* = 0.607), but not group × time interaction (*F*_1,17_ = 0.178, *p* = 0.993, η^2^*p* < 0.001), was seen for 1RM_SQUAT_. No difference was observed between groups for the absolute increase (pre- to post-intervention) (mean difference [95%CI] = -0.1 [-15.5 to 15.2 kg]; *p* = 0.992). The between-group effect size was only trivial (*d* = -0.004) (Table 3).

### Muscle endurance

For 60%1RM_BENCH_, only a significant main effect of time (*F*_1,17_ = 14.122, *p* = 0.002, η^2^*p* = 0.454), but not group × time interaction (*F*_1,17_ = 0.187, *p* = 0.671, η^2^*p* = 0.011), was found. For the absolute increase from baseline, no significant between-group difference was noted (mean difference [95%CI] = 0.5 [-1.6 to 2.5 repetitions]; *p* = 0.670). A small effect size (d = 0.20) was observed between groups (Table 3).

A significant main effect of time (*F*_1,17_ = 13.711, *p* = 0.002, η^2^*p* = 0.446), but not group × time interaction (*F*_1,17_ = 0.165, *p* = 0.690, η^2^*p* = 0.010), was observed for 60%1RM_SQUAT_. No between-group difference was noted for the absolute increase from baseline (mean difference [95%CI] = 0.7 [-2.9 to 4.4 repetitions]; *p* = 0.690). The between-group effect size was only trivial (d = 0.19) (Table 3).

### Total load lifted

**FIG. 2:**
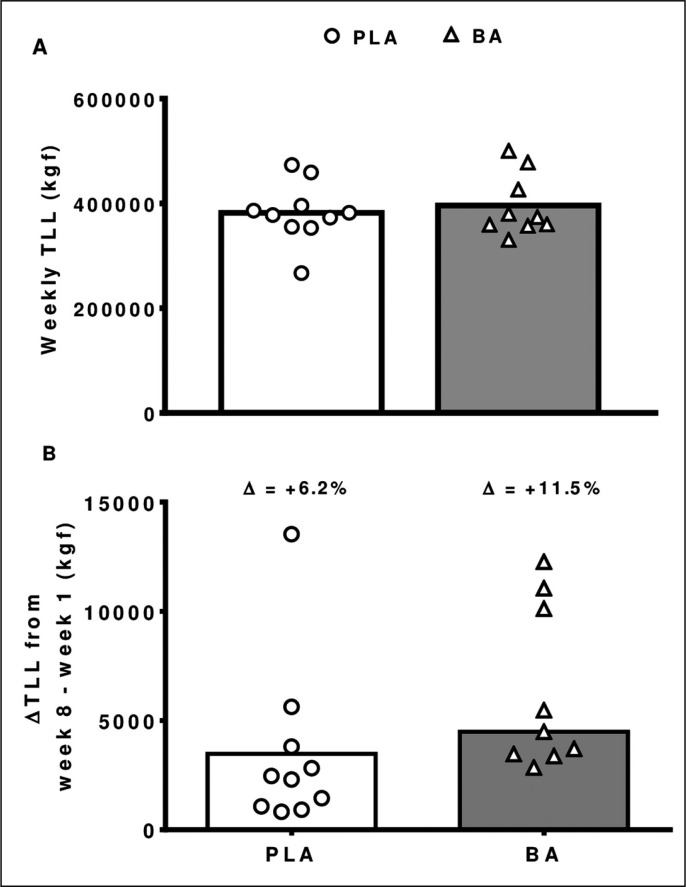
Sum of weekly total loads lifted (TLL) in the BA and PLA groups during the 8-week training period (2A). The delta Δ and relative (%) changes in TLL measured at week 8 versus week 1 (2B). All p > 0.05.

No difference between groups was observed for the sum of weekly TLL (*p* = 0.610; mean difference [95%CI] = 23978 [-6421 to 54378 kgf]) (Figure 2A) and ΔTLL week 8 - week 1 (*p* = 0.102; mean difference [95%CI] = 3067 [-17 to 6151 kgf]) (Figure 2B).

## DISCUSSION

The present study aimed to assess the chronic effects of BA supplementation on muscle strength and morphology in RT participants undertaking an 8-week training programme. Refuting the initial hypothesis, the main finding was that BA supplementation did not maximize RT-induced adaptations.

Although a significant overall training effect was noted for muscle morphology, no significant difference was detected between groups for MT_BB,_ MT_TB_ and MT_VL_. Divergent results have been reported by studies that assessed body composition outcomes with exercise plus BA supplementation combined [10, 13]. However, possible local adaptations had not been investigated yet. To our knowledge, this was the first study to assess MT outcomes following a BA supplementation and training protocol, which limits comparisons with previous investigations. Therefore, future studies assessing the possible effects of BA on MT are encouraged.

A positive training effect on 1RM strength was found from pre- to post-training, but no significant difference between groups was noted for both 1RM tests. Research to date about the effects of BA on maximal strength outcomes during an RT programme is limited. Our results corroborate previous investigations from Hoffman et al. [25] and Outlaw et al. [26]. The predominant phosphagenic nature of 1RM tests may explain the absence of effects of BA on this variable; their performance would not be limited by muscle acidosis [27]. Controversially, Maté-Munhoz et al. [28] and Hoffman et al. [12] described significant increments in 1RM following a supplementation protocol. However, differences regarding the RT protocol adopted and the use of additional ergogenic supplements (creatine in the study of Hoffman et al. [12]) during the intervention periods must be considered when attempting to explain the distinct results between the present study and the aforementioned ones. In addition, the increases in maximal strength observed by these investigations might have been an indirect effect of the increased training volume induced by BA supplementation.

Due to the buffering nature of an augmented muscle carnosine content, the initial hypothesis was that BA would induce increases in training volume, which, in turn, would result in significant strength and hypertrophic adaptations [29]. Our results corroborate findings from Bassinelo et al. [19], in which a 4-week supplementation protocol was not able to induce significant increases in the total training volume performed in both upper and lower limb exercises. Contrary to the current study, Hoffman et al. [12] reported increases in TLL in RT men for the barbell squat and bench press exercises following 10 weeks of supplementation. However, it should be noted that the TLL data from our study were reported as the sum of all exercises performed during the intervention weeks, while in the study of Hoffman et al. [12] only the TLL of 2 exercises was considered, although several exercises were performed during the training protocol. Thus, it is possible that, from an entire RT session standpoint, TLL might be less influenced by the supplementation. Increases in the TLL resulting from 3 weeks of BA supplementation were reported by the cross-over study of Hoffman et al. [25]. However, it is important to note that Hoffman et al. [25] adopted a short wash-out period (3 weeks) between conditions (with vs without supplementation), which probably did not allow muscle carnosine levels to fully return to the pre-intervention values [30], constituting a confounding factor and limiting inferences about the isolated effects of BA on training volume. Therefore, although speculative, the lack of effect of supplementation on strength and morphological adaptations observed may be attributed to the non-significant effects of BA on TLL outcomes.

For muscle endurance, our initial hypothesis was that, due to the glycolytic nature of strength endurance tests (and higher muscle acidosis) [19], BA supplementation would promote significant increases in the number of repetitions performed. Although the 8-week intervention period promoted significant increases in muscle endurance for both groups, the number of repetitions performed in the 60%1RM tests was not influenced by BA supplementation. Conflicting findings have been reported regarding BA and muscle endurance outcomes. While our results corroborate findings from Kendrick et al. [10] and Bassinelo et al. [19], other studies were able to detect additional effects on muscle endurance performance when supplementing BA [11, 26]. The characteristics of the participants [26] (gender, training experience) and the training protocol employed might help to explain these divergent results. In addition, based on the findings of Sale et al. [11] and Bassinelo et al. [19], isometric exercise tests may provide a more suitable means to test the hypothesis that an increase in muscle carnosine content (induced by BA) improves exercise capacity and performance, due to enhanced muscle buffering. Moreover, the optimal exercise intensity to enhance anaerobic demand seems to be around 45% of the maximal isometric voluntary contraction [31], which suggests that the magnitude of acidosis induced by the 60%1RM test in the current study was not sufficient to impair performance.

Exercise duration has been identified as the main factor influencing the effectiveness of BA supplementation [9]. Previous meta-analytic data have reported no difference in the effect sizes between PLA and BA groups on exercising protocols lasting < 60 seconds [32], which may help to explain the lack of effects of BA on the exercise capacity and performance in the current study. Therefore, RT protocols characterized by a much higher number of repetitions (longer time under tension) and/or with shorter rest periods might, due to increased hydrogen ion accumulation and greater muscle acidosis, derive a more meaningful ergogenic effect from BA supplementation [28].

The main limitation of the study is that no methods were used to assess participants’ muscular carnosine content, making it difficult to draw inferences about the influence of this variable on performance and morphological outcomes. In addition, it is important to note that the present results may not translate to other exercise modalities and populations, which may benefit from a supplementation protocol. Therefore, future studies with different characteristics regarding supplementation, participants’ levels and training protocols are encouraged.

## CONCLUSIONS

In conclusion, an 8-week RT programme promoted improvements in muscle size, strength and endurance capacity in RT men, but supplementation with 6.4 g of BA per day did not enhance these adaptations, relative to a PLA.
